# Amphetamine Positive Urine Toxicology Screen Secondary to Atomoxetine

**DOI:** 10.1155/2013/381261

**Published:** 2013-01-30

**Authors:** Joshua L. Fenderson, Amy N. Stratton, Jennifer S. Domingo, Gerald O. Matthews, Christopher D. Tan

**Affiliations:** ^1^Uniformed Services University of the Health Sciences, 4301 Jones Bridge Road, Bethesda, MD 20814, USA; ^2^Department of Internal Medicine, Tripler Army Medical Center, 1 Jarrett White Road, Honolulu, HI 96859-5000, USA; ^3^Department of Psychiatry, Tripler Army Medical Center, 1 Jarrett White Road, Honolulu, HI 96859-5000, USA; ^4^Department of Pharmacy, Tripler Army Medical Center, 1 Jarrett White Road, Honolulu, HI 96859-5000, USA

## Abstract

The aim of this paper is to report the first case of atomoxetine leading to false-positive urine drug screen. An otherwise healthy 27-year-old female with a history of attention deficit hyperactivity disorder (ADHD) treated with atomoxetine had an acute onset tonic-clonic seizure. On arrival to the hospital, a urine toxicological drug screen with immunochemical cloned enzyme donor immunoassay (CEDIA) was performed. Results were positive for amphetamines; however, the presence of these substances could not be confirmed with urine gas chromatography-mass spectrometry (GC-MS). She denied any illicit drug use, herbal medications, or supplements, and her other prescription medications have not been previously known to cause a false-positive result for amphetamines. While stimulant treatments for ADHD could certainly result in a positive result on urine screen for amphetamines, there have been no reports of false-positive results for amphetamines secondary to patients using atomoxetine. We implicate atomoxetine, and/or its metabolites, as a compound or compounds which may interfere with urine drug immunoassays leading to false-positive results for amphetamines CEDIA assays.

## 1. Introduction

**Figure 1:**
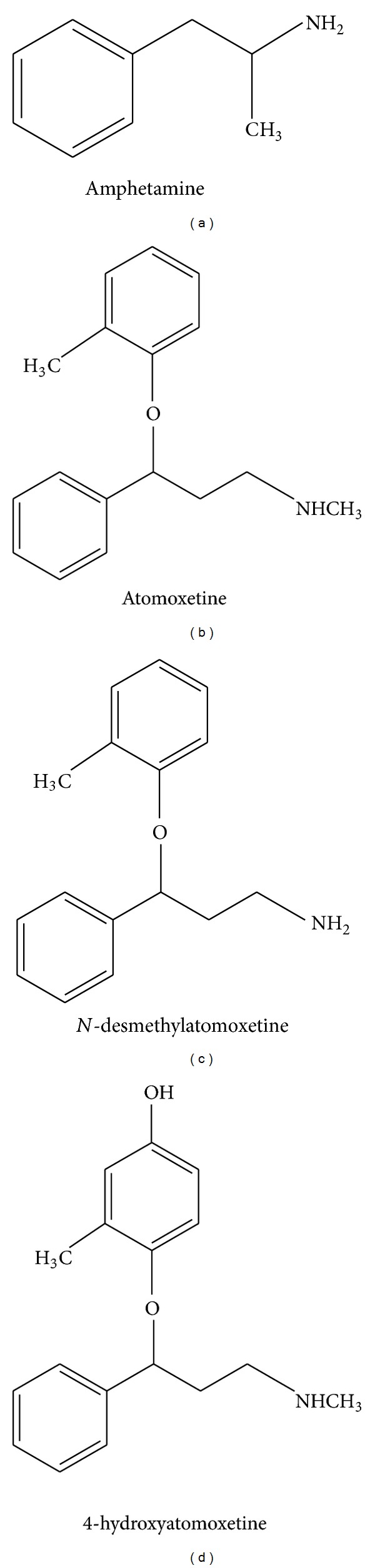
Chemical structure of atomoxetine and its main metabolites *N*-desmethylatomoxetine and 4-hydroxyatomoxetine compared with that of amphetamine [3].

Atomoxetine is classified as a selective norepinephrine reuptake inhibitor and is commonly prescribed as a nonstimulant treatment for the management of ADHD in children and adults. The drug acts at presynaptic norepinephrine transporters in neurons blocking removal of norepinephrine from the synaptic cleft through a mechanism that is not fully understood [1]. The primary oxidative metabolite of atomoxetine is 4-hydroxyatomoxetine (active metabolite but present in a much lower concentration compared to the parent drug) which is subsequently conjugated to 4-hydroxyatomoxetine-*O*-glucoronide via the cytochrome P450 CYP2D6 pathway [1–3]. To a lesser extent, atomoxetine is demethylated to *N*-desmethylatomoxetine, which is substantially less active compared to the parent drug. Structurally, atomoxetine [*N*-methyl-3-(2-methylphenoxy)-3-phenylpropan-1-amine] bears some similarities to amphetamine [1-phenylpropan-2-amine] (Figure 1) [1, 3]. Recent research also suggests that atomoxetine blocks *N*-methyl-*D*-aspartate (NMDA) receptors altering glutaminergic transmission, and this mechanism may contribute to its efficacy as a treatment for ADHD [4].

To our knowledge, neither atomoxetine nor its metabolites, have previously been reported in the literature to cause a false-positive result for amphetamines on urine drug screen. We present a case of a young female patient taking atomoxetine irregularly whose urine drug screen returned as a false-positive amphetamine result that was not confirmed. There is no evidence that the patient was taking any other substances that previously reported to interact with the amphetamines CEDIA assay, or any other explanation for these results. 

## 2. Case Summary

The patient is a 27-year-old female with a past medical history of ADHD who presented to the ER via ambulance after having two episodes of tonic-clonic movements. The first episode occurred at home and was witnessed by the patient's friend. The second episode occurred in the ambulance en route to the hospital. Upon arrival to the hospital, she was in stable condition, alert and oriented, with no visual or other neurological symptoms, and negative review of systems. The patient was being treated with atomoxetine for ADHD. Though the drug was scheduled as daily dosing, she admitted to using the medication as needed and occasionally taking more than the prescribed dose. She had taken 120 mg of atomoxetine approximately 12-hours prior to presentation, which was three times her prescribed dose and above the maximum recommended dose of 100 mg per day. Other significant medical history includes asthma and capsular contraction after recent breast augmentation. Her asthma was well controlled and did not require medications. Her capsular contraction was treated with zafirlukast 20–40 mg 1-2 times daily in addition to hydromorphone 2 mg orally and diazepam 5 mg orally to control pain and muscle spasms, respectively. She denies taking diazepam, hydromorphone, or zafirlukast within 7 days prior to presentation. She also denied the use of any OTC medications, herbal supplements, or illicit drugs. On admission, her blood pressure was 112/63 mmHg with a heart rate of 58 beats per minute, a respiratory rate of 11 respirations per minute, and an oral temperature of 96.2 degrees Fahrenheit. Her examination at presentation was normal without neurologic or focal deficits. The patient was admitted to the hospital for further diagnostic evaluation, and a CT scan revealed pituitary apoplexy with diffuse hypertensive intracerebral hemorrhage. Despite these findings, she did not receive any beta-blocker therapy as she was normotensive on presentation and throughout her admission. She had never previously been diagnosed with or treated for hypertension. Atomoxetine has been reported to cause transient hypertension, increases in baseline blood pressure, and headaches [5]. It was postulated that, in this patient, the abnormal CT findings consistent with hypertensive changes were secondary to atomoxetine associated transient hypertension. 

Further, the patient's seizures and pituitary apoplexy lead to a diagnosis of pituitary mass thought to be an adenoma. She underwent a transphenoidal resection at which point gross and histological examination of the lesion were consistent with a Rathke's cleft cyst. She tolerated the procedure well with resultant, expected diabetes insipidus that was responsive to treatment with low-dose oral hydrocortisone. Since then, she was returned to baseline function and activity without recurrent seizure activity.

As part of the workup, the patient's urine was screened for illicit drugs and toxins. Her urine was negative for tetrahydrocannabinol (THC), benzodiazepines, cocaine, and opiates. However, her urine was positive for amphetamines despite the patient denying use of any other medications or illicit drugs. A confirmatory urine GC-MS test was ordered and returned negative for amphetamine, methamphetamine, *M*-dioxyamphetamine, 3,4-methylenedioxy-*N*-methylamphetamine (MDMA), and 3,4-methylenedioxy-*N*-ethylamphetamine (MDEA). Of note, the urine GC-MS test does not directly evaluate for or confirm the presence of atomoxetine.

## 3. Discussion

Many pharmaceutical and over-the-counter (OTC) medications have been previously reported in the literature to cause a false-positive result for amphetamines on urine drug screen. Antihistamines, antipsychotics, and antidepressants are among the most well-known prescription and OTC medications that can cause false-positive urine drug screens [6]. The prescription medications known to cause false-positive amphetamine urine drug screen include fluoxetine, selegiline, ranitidine, trazodone, nefazodone, brompheniramine, phenylpropanolamine, chlorpromazine, promethazine, ephedrine, methamphetamine, and labetalol [6–9]. OTC medications are well recognized as causing a false-positive amphetamine urine drug screen include nasal decongestants, Vicks inhaler, MDMA, and pseudoephedrine [6–9]. The drug bupropion (an atypical antidepressant that inhibits norepinephrine and dopamine reuptake at the synaptic cleft) is primarily used to treat depression and smoking cessation, but may also be used off-label to treat ADHD. In a number of recent case reports, it has been implicated as an etiology of false-positive amphetamines on urine drug screen [10]. Finally, atomoxetine's major metabolites and amphetamine share some structural similarities (phenylpropan-1-amine verses phenyl-propan-2-amine). Perhaps this could be the reason for the cross-reactivity with the CDEA immunoassay.

In conclusion, the patient's medication regimen included atomoxetine, diazepam, hydromorphone, and zafirlukast. Diazepam, hydromorphone, or zafirlukast have not been reported to elicit a false-positive, for amphetamines on CEDIA immunoassay. She was not taking any additional OTC medications, herbal supplements, or illicit drugs which could interfere with urine toxicology screening. Therefore, we believe atomoxetine or its metabolites to be the cause of the false-positive test result in this patient. This highlights the first confirmed case of atomoxetine induced false-positive amphetamine on CEDIA immunoassay. This case may add to the current documentation of prescription medications known to elicit false-positive result for amphetamine on urine toxicology screen.
